# High mortality and poor treatment efficacy of immune checkpoint inhibitors in patients with severe grade checkpoint inhibitor pneumonitis in non‐small cell lung cancer

**DOI:** 10.1111/1759-7714.13187

**Published:** 2019-09-03

**Authors:** Mari Tone, Takehiro Izumo, Nobuyasu Awano, Naoyuki Kuse, Minoru Inomata, Tatsunori Jo, Hanako Yoshimura, Jonsu Minami, Kohei Takada, Shingo Miyamoto, Hideo Kunitoh

**Affiliations:** ^1^ Department of Respiratory Medicine Japanese Red Cross Medical Center Tokyo Japan; ^2^ Department of Medical Oncology Japanese Red Cross Medical Center Tokyo Japan

**Keywords:** Immune checkpoint inhibitor, interstitial lung disease, non‐small cell lung cancer

## Abstract

**Background:**

The treatment efficacy of immune checkpoint inhibitor (ICI) and clinical outcomes in patients with non‐small cell lung cancer (NSCLC) who develop severe grade checkpoint inhibitor pneumonitis (CIP) are unclear. Here, we report on the treatment efficacy of ICI and prognosis in NSCLC patients with severe grade CIP.

**Methods:**

In this retrospective cohort study, CIP severity, CIP‐related mortality, and ICI efficacy in 71 patients with advanced NSCLC treated with ICIs were evaluated. Data was obtained from the patients’ medical charts.

**Results:**

All grade and severe grade CIP were observed in 22 and 11 patients, respectively. The CIP‐related mortality rate was 22.7% (*N* = 5). An Eastern Cooperative Oncology Group (ECOG) Performance Status (PS) score of ≥2 and pre‐existing interstitial lung disease (ILD) were significantly associated with the development of severe grade CIP (*P* = 0.001 and *P* = 0.035, respectively). The median progression‐free survival (PFS) and overall survival (OS) were significantly shorter in patients with severe grade CIP than in those without severe grade CIP (PFS 1.0 month, 95% confidence interval [CI] 0.5–2.0 vs. 3.5 months, 95% CI 2.0–5.0 months, *P* = 0.003; OS 3.0 months, 95% CI 0.5–13 vs. 12.7 months, 95% CI 8.0–21.0 months, *P* = 0.011).

**Conclusion:**

CIP is a serious complication with a poor prognosis associated with high mortality. The efficacy of ICI is significantly worse in patients with severe grade CIP than in those without severe grade CIP. Whether ICIs should be administered to patients with CIP risk factors, such as an ECOG PS score of ≥2 or pre‐existing ILD, should be carefully assessed.

## Key points

### Significant findings of the study

CIP‐related morbidity was 22.7%. Median PFS and OS were significantly worse in patients with severe‐grade CIP (PFS 1.0 vs. 3.5 months, *P* = 0.030; OS 3.0 vs. 12.7 months, *P* = 0.011).

### What this study adds

CIP should be recognized as a poor prognostic predictor unlike other irAEs. ICI administration to patients with CIP risk factors, such as an ECOG PS score of ≥2 or pre‐existing ILD, should be carefully assessed.

## Introduction

The application of immune checkpoint inhibitors (ICIs) has greatly expanded and the efficacy of ICIs is clinically useful in various diseases, including lung cancer.^1^, ^2^, ^3^, ^4^, ^5^ Because the combination therapy of ICIs and cytotoxic agents can be used in non‐small cell lung cancer (NSCLC) as a first‐line treatment, the chance of receiving ICI treatment has increased, particularly in patients with NSCLC.^6^, ^7^, ^8^, ^9^

The development of immune‐related adverse events (irAEs) should be considered in patients treated with ICIs.^10^, ^11^ However, the majority of irAEs are not associated with an increased risk of death. Checkpoint inhibitor pneumonitis (CIP) is one irAE that can lead to death, particularly in patients with NSCLC.^12^ Large scale clinical trials reported 1%–5% as the morbidity rate of CIP in patients with NSCLC,^13^, ^14^, ^15^, ^16^, ^17^ whereas high morbidity rates ranging from 13.2% to 19.0% have been reported in studies in real‐world settings.^18^, ^19^ This is because the real‐world clinical studies included patients who were older, in poor general condition, or with pre‐existing interstitial lung diseases (ILDs). Additionally, previous studies have reported pre‐existing ILD and the absence of extrathoracic metastases as risk factors for CIP.^12^, ^18^

The development of irAEs, including CIP, is considered a good predictive factor for the efficacy of ICI treatment.^20^ However, patients with severe grade CIP often experience poor outcomes following ICI treatment in clinical practice. To our knowledge, to date, no study has evaluated the treatment efficacy of ICI treatment and prognosis in patients with NSCLC experiencing severe grade CIP. Here, we report on the treatment efficacy of ICI and clinical outcomes in NSCLC patients with severe grade CIP.

## Methods

### Patient selection and data collection

This retrospective cohort study included patients with stage IV, unresectable stage III, or postoperative recurrent NSCLC treated with any ICI except durvalumab at the Japanese Red Cross Medical Center between January 2016 and January 2019. Data were collected from the medical charts. For patients treated with ICIs more than once, data related to the first ICI administration were included in the current study.

The assessment and treatment of irAEs, including CIP, that occurred during the observation period in response to ICIs was based on the American Society of Clinical Oncology (ASCO) Clinical Practice Guidelines.^21^ The attending physicians diagnosed CIP clinically. When patients had clinical symptoms of cough or dyspnea and computed tomography (CT) findings of ground‐glass opacity or consolidation, several examinations were performed, such as sputum cultures, echocardiography and laboratory tests (procalcitonin, glucan endo‐1,3‐beta‐D‐glucosidase, and brain natriuretic peptide, etc) in order to exclude pulmonary infections and pulmonary edema. In addition, CIP diagnosis was occasionally made if there had been a poor response to antibiotic treatment. The efficacy of ICIs was evaluated based on overall response rate (ORR), progression‐free survival (PFS), and overall survival (OS). Response to ICI treatment was assessed by the attending physician based on the Response Evaluation Criteria in Solid Tumors version 1. PFS was defined as the time interval from ICI treatment initiation to the date of disease progression (PD) or death from any cause.

This study mainly focused on patients with severe grade CIP (grade 3 or worse) in response to ICIs. Patients were divided into groups “with severe grade CIP” and “without severe grade CIP” to evaluate the baseline characteristics, severity and mortality associated with CIP, and efficacy of ICI (ORR, PFS, and OS in both groups).

### Ethical considerations

This retrospective study was approved by the Institutional Review Board of the Japanese Red Cross Medical Center (No. 983) and registered with the University Hospital Medical Information Network (UMIN 000037184). Due to the retrospective study design and based on the Japanese ethical guidelines for clinical research, the requirement for informed consent was waived in the current study.

### Statistical analysis

To evaluate factors associated with lung injury due to ICI therapy, Fisher's exact test was used for categorical data and the Mann‐Whitney U test for numerical data. ORRs to ICIs were compared using the chi‐square test. The PFS and OS curves were generated by the Kaplan‐Meier method and compared using the log‐rank test. Because CIP is a time‐varying covariate, we performed a one‐month landmark analysis for PFS and OS to account for immortal time bias. Patients who were progression‐free or alive at one month after the initiation of ICI administration were only included in the one‐month landmark analysis for PFS or OS, respectively. A one month cutoff was identified because the median onset of severe grade CIP was one month in our study. We defined the initiation of ICI administration as time 0. Furthermore, we performed univariate analysis of PFS and OS using Cox proportional hazard regression. The descriptive statistics presented in the current study included means, frequencies, and percentages. All reported *P*‐values were two‐sided, and *P*‐values of <0.05 were considered statistically significant. All statistical analyzes were performed using EZR (Saitama Medical Center, Jichi Medical University, Saitama, Japan), a graphical user interface for R (the R Foundation for Statistical Computing, Vienna, Austria).

## Results

### Baseline patient characteristics

A total of 73 patients were treated with ICIs for advanced NSCLC at the study institution during the study period. Of the 73 patients, two who received durvalumab as maintenance therapy after chemoradiotherapy were excluded; therefore, the remaining 71 patients were included in the final analysis. The baseline characteristics of the study cohort at the time of ICI treatment are summarized in Table 1. Among the ICIs, pembrolizumab, nivolumab, and atezolizumab were administered in 21, 40, and 10 patients, respectively. There was a median of one treatment line (range 0–4) of chemotherapy preceding the ICI treatment, and 15 patients received pembrolizumab as first‐line therapy. After receiving the ICI treatment, 22 and 11 patients developed all grade and severe grade CIP, respectively. Thus, 11 patients were categorized as “with severe grade CIP” group, whereas the remaining 60 patients were categorized as “without severe grade CIP” group.

**Table 1:** Baseline characteristics of patients at the time of ICI therapy

Variables	Total (*N* = 71)	With severe grade CIP (*N* = 11)	Without severe grade CIP (*N* = 60)	*P*‐value
Age, years	69 (44–84)	65 (44–80)	69 (44–84)	0.45
Male/Female	54 (76.1) /17 (23.9)	7 (63.6)/4 (36.4)	47 (78.3)/13 (21.7)	0.29
ECOG PS 0–1/2–3	54 (76.1) /17 (23.9)	5 (45.5)/6 (54.5)	49 (81.7)/11 (18.3)	0.010
Non‐sq/sq./unknown	54 (76.1) /16 (22.5)/1 (1.4)	8 (72.7)/3 (27.3)/0 (0)	46 (76.7)/13 (21.6)/1 (1.7)	0.85
PD‐L1 expression				0.82
0%	4 (5.6)	0(0)	4 (6.7)	
1%–49%	11 (15.6)	2 (18.2)	9 (15.0)	
50%–100%	28 (39.4)	5 (45.5)	23 (38.3)	
Unknown	28 (39.4)	4 (36.3)	24 (40.0)	
Brinkman Index	720 (0–2400)	600 (0–166)	735 (0–2400)	0.38
KL‐6 (U/mL)	415 (72–33 040)	384 (223–1583)	415 (72–33 040)	0.73
SP‐D (ng/mL)	64.2 (7.2–435)	35.9 (17.2–435)	66.8 (7.2–299)	0.64
ICI				0.82
Pembrolizumab	21 (29.6)	5 (45.5)	16 (26.7)	
Nivolumab	40 (56.3)	5 (45.5)	35 (58.3)	
Atezolizumab	10 (14.1)	1 (9.0)	9 (15.0)	
Pre‐ICI treatment lines	1 (0–4)	1 (0–4)	1 (0–4)	0.62
Complicated with radiation pneumonitis	11 (15.5)	0 (0)	11 (18.3)	0.12
Complicated with other IPs†	7 (9.9)	3 (27.3)	4 (6.7)	0.035

† Other IPs included rheumatic lung disease (*N* = 2) and idiopathic interstitial pneumonitis (*N* = 5).

Data are presented as median (range) or *N* (%).

CIP, checkpoint inhibitor pneumonitis; ECOG PS, Eastern Cooperative Oncology Group Performance Status; ICI, immune checkpoint inhibitor; IP, interstitial pneumonia; KL‐6, sialylated carbohydrate antigen Krebs von den Lungen‐6; PD‐L1, programmed death‐ligand 1; SP‐D, surfactant protein‐D; sq., squamous cell carcinoma.

The Eastern Cooperative Oncology Group (ECOG) performance status (PS) score was significantly worse in “with severe grade CIP” group than that in the “without severe grade CIP” group (*P* = 0.001). There were no significant differences in tissue type, programmed death‐ligand 1 expression, levels of sialylated carbohydrate antigen Krebs von den Lungen‐6 and surfactant protein‐D, ICI type, or treatment line preceding the ICI therapy between the two groups.

Radiation pneumonitis developed in 11 patients before the ICI therapy and seven patients had other ILDs (rheumatic lung disease, *N* = 2; idiopathic interstitial pneumonitis [IIP], *N* = 5) at the time of ICI therapy. The CT pattern was probable usual interstitial pneumonitis in all five patients with IIP. The patients in “severe grade CIP” group were more frequently complicated with a pre‐existing ILD except radiation pneumonitis than “without severe grade CIP” group (27.3% vs. 6.7%, *P* = 0.035), although there was no significant difference in morbidity due to radiation pneumonitis between the two groups (0% vs. 18.3%, *P* = 0.12).

### Severity of CIP and other irAEs in response to ICI therapy

Of the 22 patients who experienced all grade CIP, 11 developed grade 3 or worse CIP in response to ICI therapy. All patients with severe grade CIP received steroid treatment for CIP according to the ASCO Clinical Practice Guidelines; seven patients received steroid pulse therapy.

**Figure 1:**
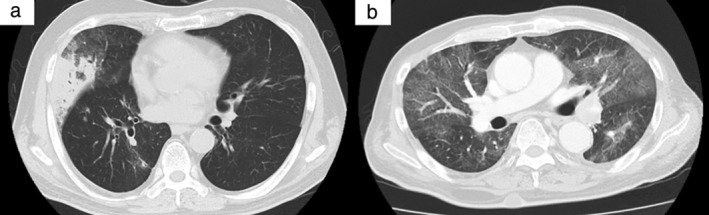
Computed tomography (CT) images of representative cases with severe grade checkpoint inhibitor pneumonitis. (**a**) Organizing pneumonia pattern. CT shows focal consolidations with surrounding ground‐glass opacities in right middle lobe. (**b**) Acute lung injury pattern. CT shows widespread ground‐glass opacities and consolidations in bilateral lungs.

Five patients died due to CIP after their chest imaging results worsened despite receiving steroid treatment for CIP. The mortality rate due to all grade CIP was 22.7%. The ECOG PS score was ≥2 in all five patients who died due to CIP, and three of the five patients had pre‐existing ILD (rheumatic lung disease and IIP). Of the five patients who died due to CIP, four patients experienced CIP during the first course of ICI therapy. None of the patients with radiation pneumonitis experienced grade 3 or worse CIP (Table 1). While CT pattern of CIP was organizing pneumonia pattern in 19 of 22 patients with all grade CIP (Fig 1a), the remaining three patients who were included in “severe grade CIP” group exhibited acute lung injury pattern (Fig 1b).

The irAEs other than CIP occurred in seven patients. Specifically, endocrine disorders (4), enterocolitis (2), hemophagocytic syndrome (1), and skin disorder (1) occurred with one patient experiencing two irAEs.

### ICI treatment efficacy and salvage chemotherapy after ICI

ICIs were administered for a median of four (range, 1–71) courses. The median number of ICI treatment courses was significantly lower in “with severe grade CIP” group than that in “without severe grade CIP” group (1 (range, 1–71) vs. 4 [range, 1–52] courses, *P* = 0.01).

The ORR to ICI tended to be lower in patients with all grade CIP than in those without CIP (18.2% vs. 37.5%) as well as in those with severe grade CIP than in those without severe grade CIP (9.1% vs. 35.6%; Table 2). Conversely, the ORR to ICI was significantly higher in patients who experienced irAEs excluding CIP than in those who did not have irAEs excluding CIP (85.7% vs. 25.4%, *P* < 0.001).

**Table 2:** Efficacy of ICI

Variables(*N* = 71)	With severe grade CIP (*N* = 11)	Without severe grade CIP (*N* = 60)	*P*‐value
Best treatment effect of ICI			0.47
CR	0 (0)	1 (1.7)	
PR	1 (9.1)	20 (33.3)	
SD	3 (27.3)	14 (23.3)	
PD	7 (63.6)	24 (40.0)	
Not comparable†	0 (0)	1 (1.7)	
ORR	9.1%	35.6%	0.20
DCR	36.4%	59.3%	0.34

† One patient had no target‐lesion which could be assessed based on the Response Evaluation Criteria in Solid Tumors version 1.

Data are presented as *N* (%).

CIP, checkpoint inhibitor pneumonitis; CR, complete response; DCR, disease control rate; ICI, immune checkpoint inhibitor; ORR, overall response rate; PD, progressive disease; PR, partial response; SD, stable disease.

**Figure 2:**
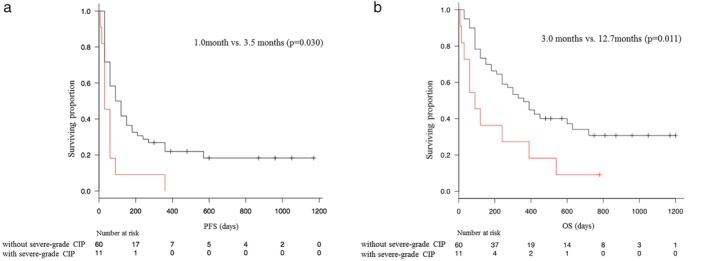
Kaplan‐Meier curves for (**a**) progression‐free‐survival (PFS) and (**b**) overall survival (OS) in patients with or without severe grade checkpoint inhibitor pneumonitis. (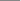) Without severe‐grade CIP and (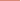) with severe‐grade CIP.

**Figure 3:**
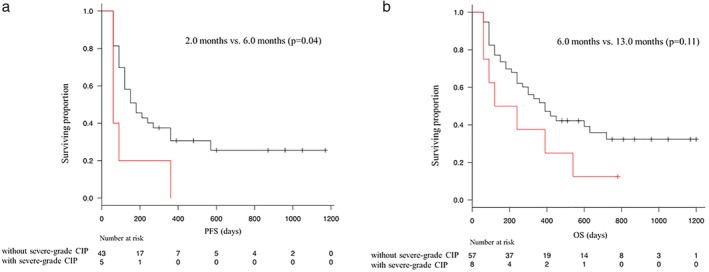
Kaplan‐Meier curves with one‐month landmark analysis for (**a**) progression‐free‐survival (PFS) and (**b**) overall survival (OS) in patients with or without severe grade checkpoint inhibitor pneumonitis. (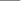) Without severe‐grade CIP and (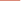) with severe‐grade CIP.

The median PFS in patients with and without all grade CIP was 2.0 (95% confidence interval [CI] 1.0–5.0) and 3.0 (2.0–5.0) months, respectively. There was no significance difference in PFS between the two groups (*P* = 0.66). Similarly, the median OS was not significantly different between the patients with and without all grade CIP (6.0 [95% CI 2.0–17.0] vs. 10.0 [7.0–15.0] months, *P* = 0.59). However, PFS and OS medians were significantly shorter in “with severe grade CIP” group than that in “without severe grade CIP” group (PFS, 1.0 [95% CI 0.5–2.0] vs. 3.5 [2.0–5.0] months, *P* = 0.003; OS, 3.0 [95% CI 0.5–13] vs. 12.7 [8.0–21.0] months, *P* = 0.011; Fig 2a,b). In addition, a one‐month landmark analysis confirmed that the median PFS was significantly different between the patients with and without severe grade CIP (2.0 [95% CI 2.0 ‐ not reached [NR]] vs. 6.0 [4.0–12.0] months, *P* = 0.04, *N* = 48). A one‐month landmark analysis showed a trend toward better median OS in the patients with severe grade CIP (6.0 [95% CI 2.0–18.0] months vs. 13.0 [12.0–21.0] months, *P* = 0.11, *N* = 65; Fig 3a,b). In the patients with other irAEs except CIP, the median PFS and OS were 19.0 (95% CI 2.0–NR) and 21.0 months (2.0–NR) months, respectively. Among the patients with mild‐grade CIP, the ORR to ICI was 27.3% and the median PFS and OS were 5 (95% CI 1–NR) and NR (95% CI 3.0–NR) months, respectively. Univariate analysis confirmed that complication with severe grade CIP and ECOG PS score of ≥2 was significantly associated with poor PFS and OS (Table 3).

**Table 3:** Cox proportional hazard regression analysis of progression‐free survival and overall survival

	PFS		OS	
	Univarate hazard ratio(95% CI)	*P*‐value	Univarate hazard ratio (95% CI)	*P*‐value
Sex	0.62 (0.34–1.10)	0.10	0.92 (0.47–1.78)	0.80
ECOG PS (0–1 vs. ≥2)	2.57 (2.40–8.79)	0.002	4.59 (2.40–8.79)	<0.001
Histology	1.15 (0.63–2.11)	0.65	1.05 (0.52–2.12)	0.89
IPs at baseline†	1.11 (0.44–2.79)	0.82	2.45 (0.95–6.34)	0.065
Radiation pneumonitis at baseline	0.65 (0.29–1.44)	0.28	0.57 (0.23–1.45)	0.24
With CIP	0.91 (0.47–1.76)	0.78	0.89 (0.46–1.72)	0.72
With severe grade CIP	2.38 (1.30–4.65)	0.011	2.35 (1.17–4.76)	0.017
With other irAEs	0.36 (0.13–1.01)	0.052	0.30 (0.07–1.24)	0.097

† Other IPs included rheumatic lung disease (*N* = 2) and idiopathic interstitial pneumonitis (*N* = 5).

CI, confidence interval; CIP, checkpoint inhibitor pneumonitis; ECOG PS, Eastern Cooperative Oncology Group Performance Status; IP, interstitial pneumonia; irAE, immune‐related adverse events; OS, overall survival; PFS, progression‐free survival.

ICI administration was discontinued in all patients in “with severe grade CIP” group, and only one of the patients could receive salvage chemotherapy after ICI. In “without severe grade CIP” group, salvage chemotherapy after ICI therapy was administered in 23 of 50 patients who discontinued the ICI therapy. The proportion of patients receiving salvage chemotherapy after ICI was significantly different between the two groups (9.1% and 38.3% in “with severe grade CIP” and “without severe grade CIP” groups, respectively; *P* = 0.024).

## Discussion

The current retrospective cohort study, including 71 patients with advanced NSCLC who received ICI therapy, revealed the severity and high mortality of CIP in response to ICI therapy. Furthermore, we found that the median PFS and OS were significantly worse in patients with severe grade CIP than those without severe grade CIP.

Our analyses revealed that 50.0% of all grade CIP cases were grade 3 or worse and that the severe grade CIP morbidity rate was 15.5% in the entire study cohort. Importantly, the CIP‐related mortality rate was 22.7% in the current study. The CIP morbidity rates were 1%–5% and 13.2%–19.0% in large scale clinical trials^13^, ^14^, ^15^, ^16^, ^17^ and studies in real‐world settings,^18^, ^19^ respectively. Similarly, the reported severe grade CIP incidence rates were 1%–3% and 3.4%–4.2% in large scale clinical trials^13^, ^14^, ^15^, ^16^, ^17^ and real‐world clinical studies,^18^, ^19^ respectively. Furthermore, although mortality related to CIP was not observed in large scale clinical trials,^13^, ^14^, ^15^, ^16^, ^17^ the CIP‐related mortality rate ranged from 12.8% to 18.2% in real‐world clinical studies.^18^, ^19^ Patients who are elderly, in poor clinical condition, or with pre‐existing ILDs are usually excluded from large scale clinical trials. However, many patients with these conditions are considered for ICI therapy in real‐world settings. In the current study, the incidence rate of severe grade CIP was significantly higher in the patients with worse ECOG PS scores and pre‐existing ILDs, except radiation pneumonitis compared with the other patients. Poor clinical condition and pre‐existing IIP, or rheumatic lung disease may be risk factors for the development and severity of CIP. Moreover, ECOG PS scores in all patients who died due to CIP were two or worse, and 60% of those patients had pre‐existing ILDs (rheumatic lung disease and IIP). These results suggest that these two factors may also be associated with high CIP‐related mortality and ICI therapy should be avoided in patients who have these factors. Additionally, salvage chemotherapy after ICIs could not be administered to most patients with severe grade CIP because their ECOG PS scores worsened after the development of CIP, and salvage chemotherapy could lead to CIP relapse. The fact that most patients with severe grade CIP cannot receive salvage chemotherapy after ICIs may also be a risk factor for their poor prognosis.

The treatment efficacy of ICIs in patients with severe grade CIP was worse than that those without severe grade CIP in the current study. The ORR to ICI tended to be lower and the median PFS and OS were significantly worse in the patients with severe grade CIP than in those without severe grade CIP. In addition, a one‐month landmark analysis and univariate analysis of PFS and OS supported these results. A previous study reported that the development of irAEs was a good predictor of survival outcomes in patients with NSCLC treated with nivolumab.^21^ The authors also showed that CIP was a good predictor of survival outcomes in patients with NSCLC. Another study showed that the ORR to nivolumab was higher in patients with pre‐existing ILD than in those without pre‐existing ILD^22^; however, the results of the present study contradicted the findings of the previous one. The difference between this and previous studies was to classify patients with CIP according to severity. Our results suggest that severe grade CIP as a complication of ICI therapy was a predictor of poor treatment efficacy of ICIs. We should recognize that poor prognosis is predicted based on the development of severe grade CIP, whereas good prognosis is predicted based on the development of other irAEs, except CIP.

The limitations of this study include the small sample size. Additionally, this was a retrospective study performed at a single institution including a heterogenous cohort of patients treated with various ICIs, and those who were administered ICIs as first‐ or later‐line treatment. Comparison of the patient characteristics in the present study with those of the previous ones suggests a significant difference in race. All patients in the present study were Japanese, and treatment‐related pneumonitis has been reported to be common in Japanese patients with lung cancer.^23^, ^24^ A large scale prospective cohort study should be conducted to further elucidate the prognosis of CIP in patients with NSCLC.

In conclusion, CIP is a serious complication with a poor prognosis in patients with NSCLC undergoing ICI therapy because pneumonitis‐related death has been observed. Moreover, the efficacy of ICI treatment was significantly worse in patients with severe grade CIP than those without severe grade CIP. These results clearly illustrate that whether ICIs should be administered to patients with CIP risk factors such as an ECOG PS score of ≥2 and pre‐existing ILD must be carefully assessed.

## Disclosure

The authors declare they have no conflict of interest.
